# Sex Differences in Arteriovenous Fistula Failure: Insights from Bioinformatics Analysis

**DOI:** 10.3390/jcdd10010003

**Published:** 2022-12-22

**Authors:** Ke Hu, Yiqing Li, Yi Guo, Peng Cheng, Yuxuan Li, Chanjun Lu, Chuanqi Cai, Weici Wang

**Affiliations:** 1 Department of Vascular Surgery, Union Hospital, Tongji Medical College, Huazhong University of Science and Technology, Wuhan 430000, China; 2 Clinic Center of Human Gene Research, Union Hospital, Tongji Medical College, Huazhong University of Science and Technology, Wuhan 430000, China; 3 Cardiovascular Center, Liyuan Hospital, Tongji Medical College, Huazhong University of Science and Technology, Wuhan 430000, China; 4 Department of Nephrology, Taihe Hospital, Affiliated Hospital of Hubei University of Medicine, Shiyan 442000, China

**Keywords:** sex differences, AVF failure, bioinformatics

## Abstract

(1) Background: Arteriovenous fistulas (AVFs) are the preferred access for hemodialysis. Unfortunately, about 60% of patients, especially female patients, fail to receive normal dialysis within one year after surgery because of AVF failure. However, the underlying mechanisms caused by sex differences in AVF failure remain unclear. (2) Methods: We performed analysis of DEGs and functional analysis with the dataset GSE119296 to reveal the biology underlying AVF failure. Immune responses were calculated using CIBERSORT. A protein–protein interaction network and hub gene were constructed using STRING and stepwise identification of potential drugs was performed online. (3) Results: Functional analysis showed that extracellular matrix reprogramming and PI3K-AKT pathway enrichment were significant in both male and female patients. COL1A1 was the hub gene in male patients, whereas CDK1 was the hub gene in female patients. Immune responses including γδ-T cells and mast cells are activated in female patients while no significant differences were noted in the male group. (4) Conclusions: In this study, we used a series of mature and recognized bioinformatic strategies to determine the following items: (1) Reveal the pathogenesis of AVF failure through HUB genes and signaling pathways between the different sexes. (2) Determine the relationship between sex differences in AVF failure and immune abnormalities. (3) Search for relevant sex-specific drugs targeting AVF failure.

## 1. Introduction

Arteriovenous fistulas (AVFs) were introduced over half a century ago and have been used extensively as the preferred choice to provide vascular access for patients with end-stage renal disease (ESRD) undergoing hemodialysis. Despite growing recognition and attention given to the importance of ESRD in health care, worldwide, over 1.5 million patients suffer from this disease and this population will continue to grow [1,2]. Globally, hemodialysis is the main modality of renal replacement therapy for more than 70% of patients, and all patients require different types of vascular access to perform the procedure [3,4]. In comparison to other modes of vascular access, AVFs were recommended by the Fistula First Initiative as the first choice to satisfy renal replacement therapies (RRT) due to their better long-term patency rate, lower probability of complications, and lower health care costs [5,6,7,8].

Unfortunately, up to 60% of patients fail to use AVF within six months after creation [4]. Failure of the outward maturation of the AVF and narrowing of inward remodeling due to intimal hyperplasia lead to complications in dialysis. AVF patency is related to numerous variables, including age, sex, underlying metabolic disease, body mass index, surgeon technique, and postoperative care [4]. Although these factors show considerable differences between different regions and populations, an increasing number of basic studies have revealed common molecular mechanisms that mediate AVF failure [9]. Invasive intimal hyperplasia is the most important cause of AVF failure and is histologically characterized by constriction of the lumen and function of the outflow tract caused by the accumulation of large numbers of contractile smooth muscle cells, fibroblasts, immune cells, and extracellular matrix [1,10,11]. The occurrence of intimal hyperplasia is closely related to biological pathways such as inflammation, hypoxia, oxidative stress, and shear stress [12]. The continuous deepening of related mechanism research has greatly improved our understanding of the occurrence and development of AVF, and provided new molecular targets and hope for intervention of AVF failure.

Notably, in recent years, the role of sex differences in vascular diseases has gradually received attention. The effects of sex differences on cardiovascular diseases are reflected in many diseases. For example, myocardial infarction was significantly more frequent in men than in premenopausal women [13]. More younger men were observed to have hypertension than younger women, whereas the opposite trend was observed in the older population [14]. Women with coronary artery disease had a later onset and correspondingly less severe symptoms [15]. Sex differences also exist in hemodialysis vascular access; compared with men, AVF maturation rates are lower in women, take longer to mature, and have a worse prognosis [16,17]. Women also require more frequent salvage surgeries for AVF strictures [18]. The different outcomes in cardiovascular diseases caused by sex differences may be ascribed to the changes in metabolism, the activation of hormones and their receptors, and the structural differentiation of vital target organs; however, the exact mechanisms remain unclear [13,19].

Several hypotheses have attempted to explain the sex differences in AVF maturation rates, including smaller mean vessel diameters in women, decreased vasodilation capacity, and insufficient outward remodeling due to estrogenic anti-inflammatory effects [16,20]. However, the specific molecular biological changes and subsequent effects caused by sex differences in the development of AVF are not clear. Bioinformatics analysis technology comprehensively analyzes the organization and integration of data, and its main goals are the screening of important differential genes in the occurrence and development of diseases, and the enrichment of related signaling pathways. It has been successfully applied to the study of numerous diseases, with some promising results [21,22,23,24].

The rapid development of intraluminal balloon dilatation technology has greatly reduced the trauma of secondary surgery for AVF patients. At the same time, the number of traditional open surgeries has also sharply declined, which acts as the main obstacle in sample collection. Martinez et al. compared the RNA expression of pre-access (native) veins and AVFs with distinct maturation outcomes through next generation sequencing for the first time in 2019 [25]. This study ameliorates the current dilemma of lack of sequencing datasets of AVF and provides a basis for further identification of potential molecular markers of AVF failure. With the increasing popularity of genomics research, some studies have attempted to explore the molecular mechanisms and core signaling pathways affecting AVF failure using bioinformatics methods without considering gender factors; the specific impact of sex differences in this disease remains unclear [22,26,27].

In this study, we used a series of mature and recognized bioinformatic strategies to determine the following items: (1) reveal the pathogenesis of AVF failure through HUB genes and signaling pathways between different sex; (2) determine the relationship between sex differences in AVF failure and immune abnormalities; (3) search for relevant sex-specific drugs targeting AVF failure.

## 2. Results

### 2.1. Characteristics of Patients and Validation of the Datasets

**Figure 1:**
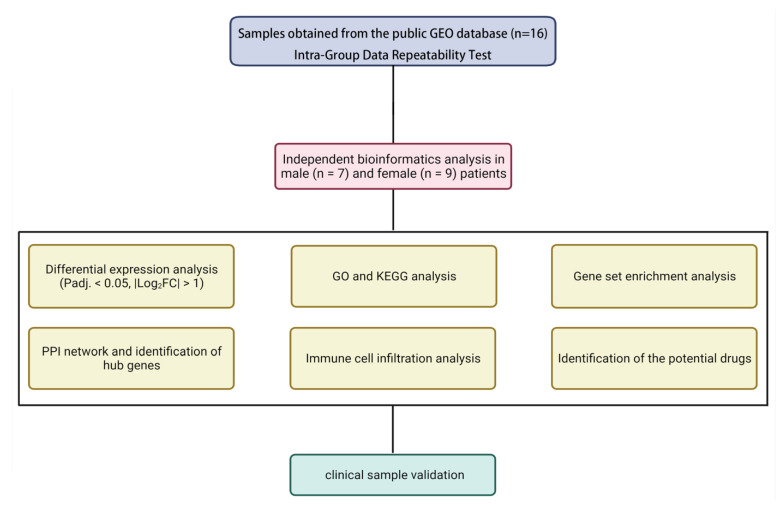
The flowchart of this research analysis.

In this study, two large types of samples from different periods were included in our selected dataset, GSE119296: native vein (stage 1) and anastomotic AVF samples (stage 2). The main focus of this research was to study the differences in the pathological evolution of stenosis after AVF remodeling between men and women. Therefore, we chose the native vein from stage 1 as the control group and the subsequent venous outflow tract specimens with stenosis as the experimental group from seven men and nine women to analyze the genetic difference, enriched function, hub genes, and immune cell infiltration, as shown in the flow chart in Figure 1. The control vein and AVF failure subsets in both experimental groups were similar in terms of patients’ baseline characteristics (Supplementary Table S1).

**Figure 2:**
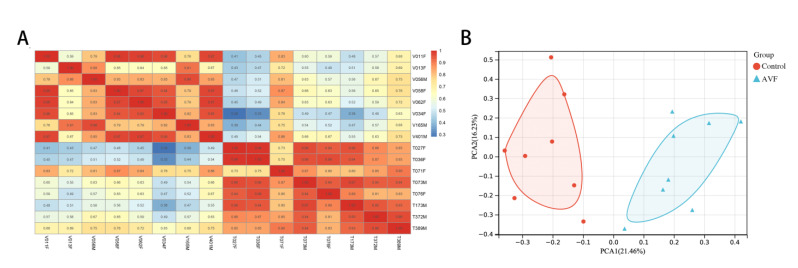
Characteristics of patients and validation of data from GSE119296. (**A**) The heatmap shows the correlation between intra-group data. (**B**) The selected samples from the dataset were analyzed using principal component analysis (PCA).

To further validate the repeatability of intra-group data, we used the Pearson correlation test between different samples and applied principal component analysis (PCA) to examine the potential association between the AVF and control samples. The heatmap presented according to the Pearson correlation test indicated a strong correlation between the control and AVF samples (Figure 2A). Thereafter, PCA was performed on the experimental and control groups, and the data were well-reproduced within the groups. The distance between the AVF samples in both PC1 and PC2 was similar to the distance between the control samples (Figure 2B).

### 2.2. Identification of DEGs

**Figure 3:**
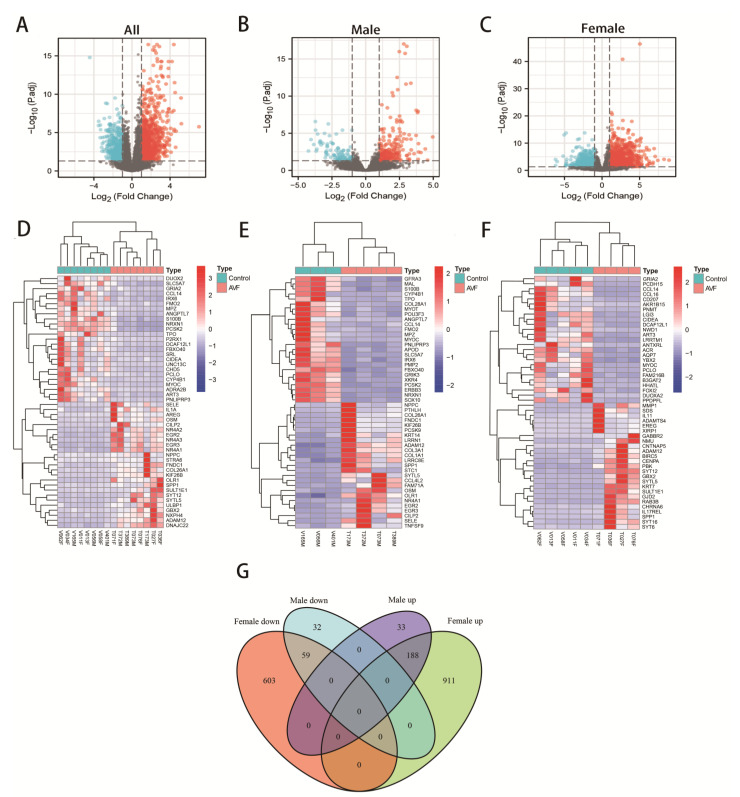
Identification of non-specific, male-specific and female-specific DEGs and Venn plots. (**A**) Volcano plot showing the non-specific DEGs. (**B**) Volcano plot showing the male-specific DEGs. (**C**) Volcano plot showing the female-specific DEGs. (**D**) Cluster heatmap of the non-specific DEGs. (**E**) Cluster heatmap of the male-specific DEGs. (**F**) Cluster heatmap of the female-specific DEGs. (**G**) Venn plots drawn for the upregulated and downregulated DEGs with male and female specificity.

In this study, we explored both integral- and sex-specific DEGs in the corresponding samples. We screened 1826 DEGs containing 1132 upregulated genes and 694 downregulated genes in eight venous tissues of AVFs compared with eight native veins in all hemodialysis patients. A volcano plot (Figure 3A) was constructed to visualize the screened DEGs. Meanwhile, 312 DEGs containing 221 upregulated genes and 91 downregulated genes were screened in four venous tissues of AVFs compared with three native veins in male hemodialysis patients, and 1761 DEGs containing 1099 upregulated genes and 662 downregulated genes were screened in four venous tissues of AVFs compared with five native veins in female hemodialysis patients. Corresponding volcano plots (Figure 3B,C) were constructed to visualize the screened DEGs. The top 50 upregulated and downregulated non-specific, male-specific, and female-specific genes are shown in the heatmaps (Figure 3D–F). Venn plots were drawn for the upregulated and downregulated DEGs with male and female specificity, as shown in Figure 3G. A total of 188 genes were upregulated, and 59 genes were downregulated in both male and female patients. In addition to 33 male-specific elevated genes, 911 female-specific elevated genes, 32 male-specific downregulated genes, and 603 female-specific downregulated genes were identified.

### 2.3. Gene Ontology and KEGG Pathway Enrichment Analysis

**Figure 4:**
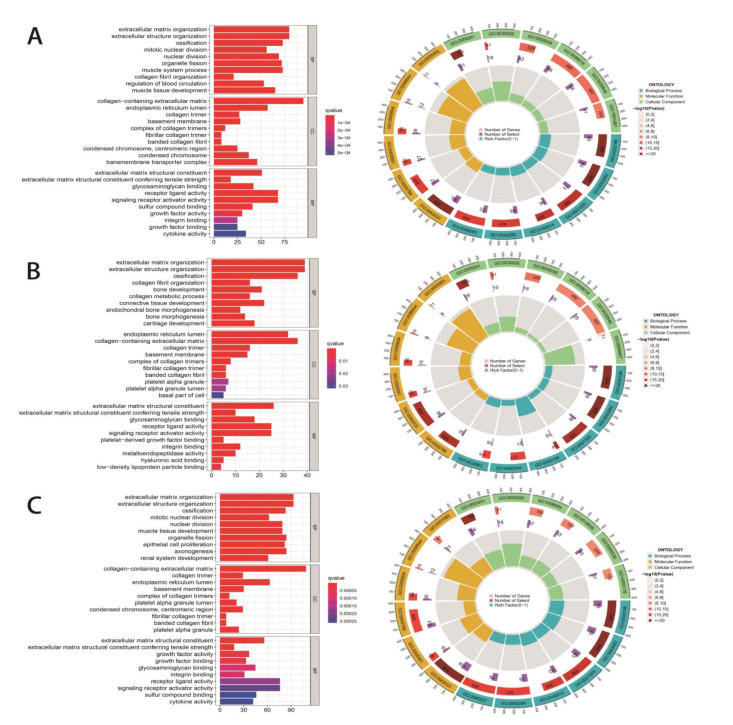
Gene ontology analysis in different sexes. (**A**) Gene ontology analysis of the non-specific DEGs. (**B**) Gene ontology analysis of the male-specific DEGs. (**C**) Gene ontology analysis of the female-specific DEGs.

**Figure 5:**
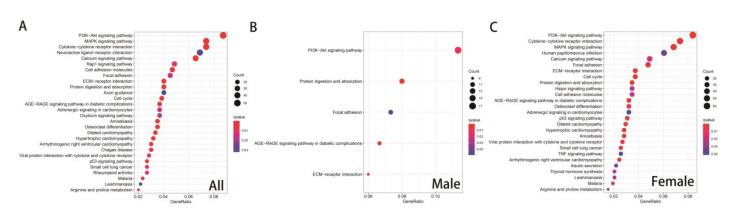
KEGG pathway enrichment in different sexes. (**A**) KEGG pathway enrichment of non-specific DEGs. (**B**) KEGG pathway enrichment of male-specific DEGs. (**C**) Gene ontology analysis of female-specific DEGs.

GO and KEGG enrichment analyses were performed based on DEGs between the native veins and the AVF group. To discover whether there were differences in sex, integral and sex-specific DEGs were analyzed. According to GO analysis, extracellular matrix-associated genes, including those involved in extracellular matrix organization, extracellular structure organization, and collagen fibril organization, were significantly different. Furthermore, these genes showed even more dramatic differences in the male group than in the female group (Figure 4A–C). In the KEGG pathway enrichment analysis, inflammatory and matrix-associated genes showed a significant difference (Figure 5A). Moreover, some differences in DEGs were larger in males than in females, such as those in genes involved in the PI3K-Akt pathway, protein digestion and absorption, focal adhesion, AGE-RAGE signaling pathway in diabetic complications, and ECM-receptor interaction (Figure 5B,C). Hence, we hypothesized that sex-specific DEGs could be the reason for the different clinical outcomes after AVF remodeling between men and women.

### 2.4. Gene Set Enrichment Analysis

**Figure 6:**
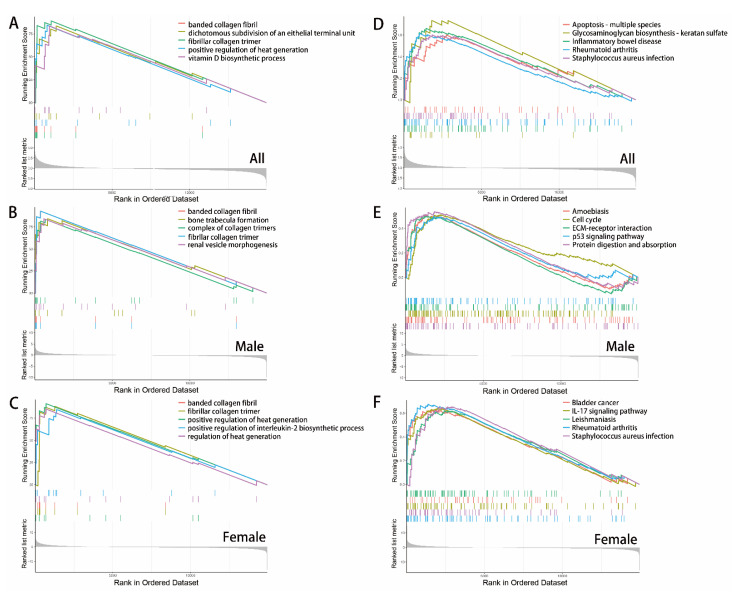
Gene set enrichment analysis in different sex. (**A**) GO gene sets enriched in AVF patients. (**B**) GO gene sets enriched in male AVF patients. (**C**) GO gene sets enriched in female AVF patients. (**D**) KEGG gene sets enriched in AVF patients. (**E**) KEGG gene sets enriched in male AVF patients. (**F**) KEGG gene sets enriched in female AVF patients.

To explore whether the identified genes caused different biological features, GSEA was performed to illustrate the enrichment maps between patients in different groups, based on distinct GO and KEGG gene sets. GSEA revealed that synthesis of collagen fibers and some inflammatory pathways were enriched in AVF patients (Figure 6A–E). The banded collagen fibril, dichotomous subdivision of an epithelial terminal unit, fibrillar collagen trimer, positive regulation of heat generation, vitamin D biosynthetic process, rheumatoid arthritis, and S. aureus infection were enriched in the integral group (Figure 6A,D). Regarding sex differences, ECM-receptor interaction, protein digestion, and absorption were enriched in the male group (Figure 6B,E), while positive regulation of IL-2 and IL-17 signaling pathways was enriched in the female group (Figure 6C,F).

### 2.5. Construction of the PPI Network and Selection of Hub Genes

**Figure 7:**
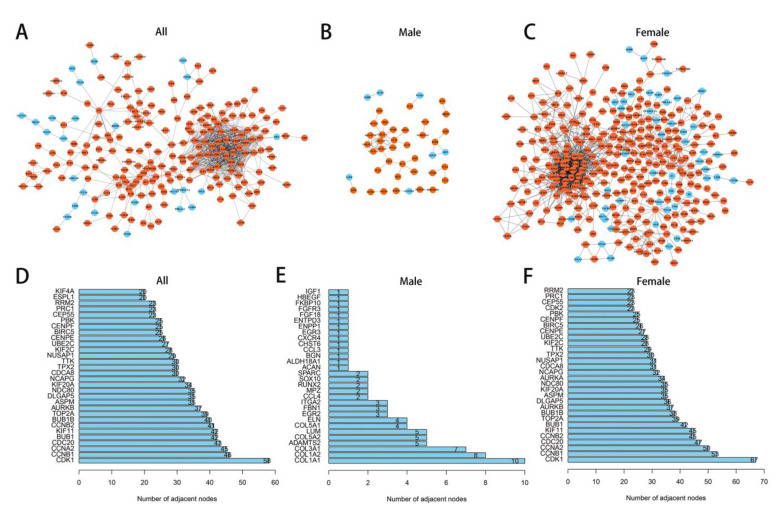
Protein–protein interaction (PPI) network and hub genes in different sex. (**A**) PPI network in AVF patients. (**B**) PPI network in male AVF patients. (**C**) PPI network in female AVF patients. (**D**) Hub genes in AVF patients. (**E**) Hub genes in male AVF patients. (**F**) Hub genes in female AVF patients.

To explore the interplay among DEGs in different groups, PPI networks were built using the STRING tool with confidence >0.9 as the cut-off criterion (Figure 7A–C). Furthermore, according to the topological property analysis of the PPI network, several nodes had a higher connectivity degree, with a degree ≥5 set as the criterion. CDK1, CCNB1, and CCNA2 in all patients; COL1A1, COL1A2, and COL3A1 in male patients; and CDK1, CCNB1, and CCNA2 in female patients were the remarkable nodes as they had the most connections with other nodes (Figure 7D,E).

### 2.6. Signature of Immune Cell Infiltration

**Figure 8:**
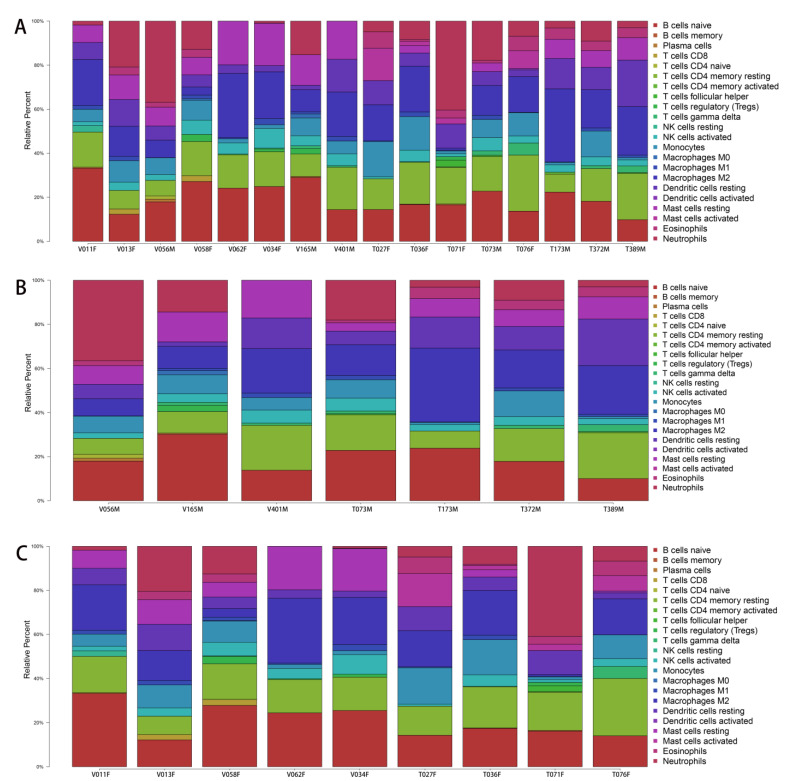
Bar charts showing the proportions of infiltrated immune cells. (**A**) Bar charts of immune cell infiltration in AVF patients. (**B**) Bar charts of immune cell infiltration in male AVF patients. (**C**) Bar charts of immune cell infiltration in female AVF patients.

**Figure 9:**
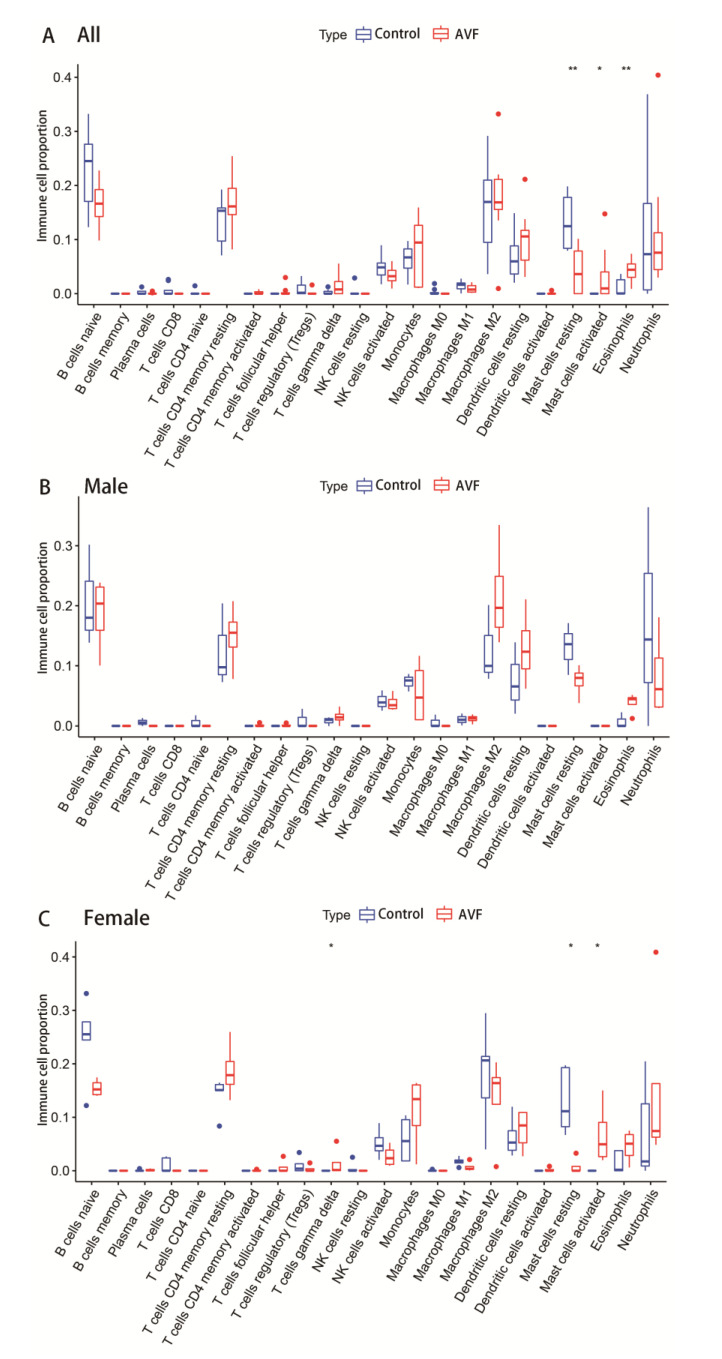
Box plots demonstrating the difference of infiltrated immune cells. (**A**) Box plot of immune cell infiltration differences in AVF patients. (**B**) Box plot of immune cell infiltration differences in male AVF patients. (**C**) Box plot of immune cell infiltration differences in female AVF patients. Significant differences are indicated by * *p* < 0.05 ;** *p* < 0.01 .

Because of the influence of the local immune environment, venous stenosis after PTA could be associated with the immune system. Therefore, immune cell infiltration was analyzed to verify the distinction between the two sexes. According to the results, the bar chart shows the proportions of infiltrated immune cells (Figure 8A–C). Based on the mRNA expression of immune-specific genes, the activities of immune cells in AVFs and native veins were quantified. In the integral group, activated eosinophils and mast cells became more efficacious than resting mast cells (Figure 9A). There was no significant difference between the male and female groups (Figure 9B). In contrast, in the female group, with the same trend as the integral group, gene expression of gamma delta T cells and activated mast cells was higher in AVF than in the control, and resting mast cells showed lower expression (Figure 9C).

### 2.7. Validation of the Hub Genes in Clinical Samples

**Figure 10:**
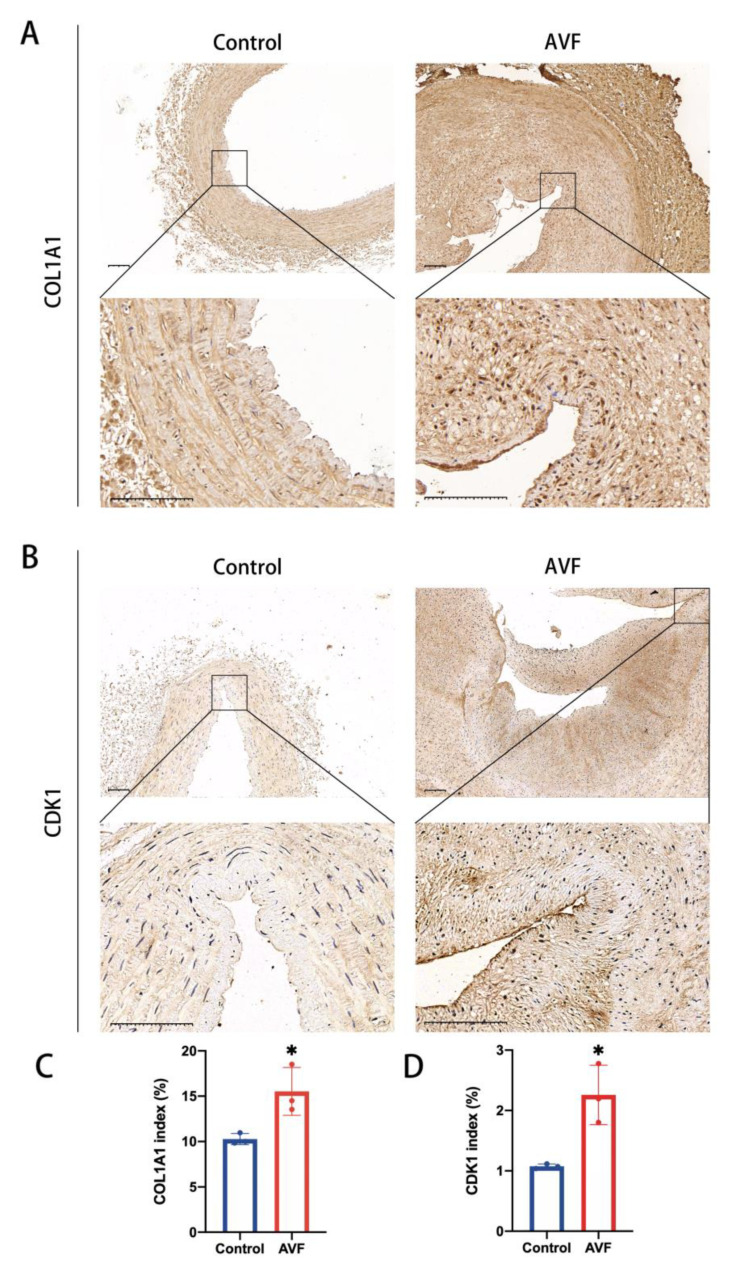
Validation of hub genes in clinical samples. (**A**) Immunohistochemical staining for COL1A1 in the native vein and AVF venous sample of a male CKD patient (N = 3). (**B**) Immunohistochemical staining of CDK1 in the native vein and AVF venous sample of a male CKD patient (N = 3). (**C**) The quantitative analysis of COL1A1 in the native vein and AVF venous sample of a male CKD patient. (**D**) The quantitative analysis of CDK1 in the native vein and AVF venous sample of a female CKD patient. Cells staining brown are positive for COL1A1 and CDK1. Two-sample *t* test was performed. Each bar represents mean ± SD. Significant differences are indicated by * *p* < 0.05. Scale bar is 100 μm. COL1A1 indicates collagen type I alpha 1; AVF, arteriovenous fistula; CDK1, cyclin-dependent kinase 1.

We examined the protein levels of hub genes in male and female chronic kidney disease (CKD) patients using clinical samples. The results showed that AVF venous samples had higher protein levels of COL1A1 in male patients (Figure 10A,C). CDK1 expression was higher in female AVF venous samples than that in native veins (Figure 10B,D).

### 2.8. Identification of the Potential Drugs

DGIdb was applied to determine the potential drugs or molecular compounds through hub genes that target AVF failure in male and female patients, as shown in the drug–gene interaction table (Supplementary Table S2). In male patients, a total of 7 target genes and 63 drugs were predicted, and in female patients, a total of 134 genes and 312 drugs were predicted. These drugs mainly include chemotherapeutic drugs that inhibit cell proliferation, as well as flavonoids and hormones. Subsequently, we searched for the drugs mentioned in the list and summarized the research results that have been obtained so far (Table 1).

## 3. Discussion

AVF has become the preferred type of vascular access for hemodialysis in patients with ESRD owing to its superior patency rate and fewer complications [1,12]. Outflow vein stenosis, caused by uncontrolled intimal hyperplasia, is a major cause of AVF failure. In contrast to surgically formed arteriovenous endovascular fistulas, spontaneously formed arteriovenous malformations share many similarities with AVF failure in the progression of the disease, especially in fistula lesions in children with cerebral venous malformations [39]. During the transition from the blood supply artery to the lesion, then to the draining vein, the vessel exhibits dysregulation of differentiation and severe dilatation remodeling [40]. Several studies have shown that in addition to mutations in genetic loci due to familial inheritance, related pathways including inflammation, growth factor receptor activation, vascular differentiation, and oxidative stress play an important role in the development of the disease [41,42,43,44]. Current studies on the mechanisms of AVF also confirm the importance of growth factors, inflammation, extracellular matrix, and oxidative stress, but the specific mechanisms of AVF failure remain poorly understood [45,46,47].

Clinical practice shows that the prognosis of female patients with AVF is worse than that of male patients [16]. Women take longer to mature and have higher rates of non-maturation compared with men [17]. Identifying the mechanisms and changes in vascular remodeling may suggest sex-specific vascular therapies to improve AVF success.

With the increasing popularity of genomics research, some studies have attempted to explore the molecular mechanisms and core signaling pathways affecting AVF intimal hyperplasia using bioinformatics methods [22,26,27]. Several studies have compared the differences between AVF outflow veins and normal veins in hemodialysis patients from different perspectives by analyzing GSE39488 microarray data from the GEO database. The differential genes between the two groups were mainly involved in cell proliferation, vascular remodeling, and inflammation-related biological functions. Unfortunately, owing to the small number of cases in this dataset, a detailed comparison of the effect of sex differences on AVF failure was difficult to achieve. In contrast, because the Agilent-026652 Whole Human Genome Microarray was chosen in this study, its detection sensitivity and reproducibility are still inferior to the current mainstream next-generation sequencing technology. In a subsequent study, Martinez et al. collected clinical venous tissue samples from patients who underwent AVF establishment surgery at Jackson Memorial Hospital and University of Miami Hospital from 2014 to 2017 and underwent next-generation sequencing for the first time. The samples were collected at two different stages. First-stage samples were obtained from the native vein used to establish the AVF during the initial surgery. Second-stage samples were obtained from the outflow tract near the proximal anastomotic area of both mature and failed arteries and veins undergoing transposition or salvage surgery. In the process of analyzing first-stage samples of native veins, researchers found that some inflammatory genes played an important role in influencing the outcome of AVF failure or maturation, and clarified the co-localization relationship between these genes and smooth muscle cells. Surprisingly, when analyzing the second-stage outflow vein samples from the mature or failed AVF, a total of 53,360 distinct RNA features were detected using RNA-seq, and only two genes were differentially expressed in the failed AVF compared with those in mature AVF (after adjusting for sex-specific expression differences). This study demonstrated that the reconstructed mature or failed AVF samples were highly similar in terms of the transcription process, and the differences were not as obvious. A possible reason is that the factors that determine the outcome of AVF maturation are already present at an earlier time before surgery, or that more decisive factors are reflected in post-transcriptional protein modifications. Therefore, the focus of research on AVF failure should be on the process of venous remodeling and transformation of native veins.

In the present study, by comparing the sequencing data of native veins and failed outflow vein samples, we aimed to determine the similarities and differences caused by sex factors in the process of vein remodeling through bioinformatics methods and predicting specific targeted therapeutic drugs. During the process of identification of differentially expressed genes, we found that male-specific DEGs were lower than female-specific DEGs, which demonstrated women experience more complicated changes during venous remodeling. Stepwise GO functional analysis showed that extracellular matrix reprogramming and PI3K-AKT pathway enrichment were both significant in male and female patients. Extracellular matrix reprogramming is significant for mature arterial structures in the venous limb of AVF when adapting to the arterial environment by increased diameter flow conductance and wall thickening. Extracellular matrix components are expressed in various patterns during AVF maturation [48,49]. The PI3K-AKT signaling pathway is involved in multiple functions, including extracellular matrix formation, wall thickening remodeling, immune system activation, and inflammation reduction [50]. Furthermore, it often performs contrasting functions in the AVF process. Dardik et al. showed that inhibition of the AKT-related pathway can alter venous remodeling and improve patency during AVF maturation [51]. Thus, inhibition of mitochondrial fission can also reduce intimal hyperplasia in AVF via the PI3K-AKT pathway [52]. In the process of comparing the KEGG signaling pathways, we found that the top five pathways enriched in female patients were the PI3K-Akt signaling pathway; cytokine and cytokine receptor interaction; MAPK signaling pathway; human papillomavirus infection; and calcium signaling pathway. The top five in male patients were protein digestion and absorption; AGE-RAGE signaling pathway in diabetic complications; the ECM-receptor interaction; the PI3K-Akt signaling pathway; and focal adhesion. The activation of the calcium signaling pathway in vascular smooth muscle cells (VSMCs) not only plays an important role in the occurrence and development of myogenic vasoconstriction, but also participates in vasoconstriction by neurohumoral mediators and other mechanical stimuli [53]. Increased intravascular pressure depolarizes VSMC membrane and activates voltage-dependent calcium channels (VDCCs), leading to Ca 2+ influx [54]. In addition, the activation of calcium pathway has been proved to promote VSMC proliferation and migration in many studies [55,56]. Therefore, regulating the calcium signaling pathway may be one of the important therapeutic means for female AVF. Notably, the AGE-RAGE signaling pathway was ranked high in male patients. In vascular diseases, the formation of AGE leads to cross-linking of collagen molecules with each other and with circulating proteins, which contributes to the development of atherosclerotic plaques [57,58]. In addition, AGEs bind to RAGE and induce reactive oxygen species (ROS) production through activation of NADPH oxidase and NF-κB signaling pathways. Reactive oxygen species (ROS) have been shown to cause smooth muscle proliferation and intimal hyperplasia [10,59]. Therefore, blocking the AGE-RAGE signaling pathway may have great potential significance for the treatment of AVF failure. Subsequently, we explored the hub genes in patients of different sexes. COL1A1 was the hub gene in male patients, whereas CDK1 was the hub gene in female patients. Type I collagen expression is associated with radial artery elasticity dysfunction in ESRD patients. Uremic toxins in patients with CKD can induce a phenotypic switch in rat aortic VSMCs, which increases type I collagen secretion and leads to extracellular matrix remodeling. CDK1, a cell-cycle associated kinase, is involved in VSMC proliferation, which is significant in intimal hyperplasia during AVF maturation [60].

The role of the immune microenvironment in diseases has received increasing attention in recent years. Agrawal et al. revealed that immune cell enrichment in AVF vessels is associated with an immune response during AVF creation and maturation [61]. In addition, pro- and anti-inflammatory immune cells, including macrophages, dendritic cells (DCs), T cells, and T-regulatory (Treg) cells, play a significant role in inflammation in AVF maturation failure. Here, we also analyzed the difference in immune cell infiltration between men and women, and found that there was no significant difference in the male group, while in the female group, γδ T cells, activated mast cells, and resting mast cells showed a significant difference. Mast cells, found in nearly all vascularized tissues, are a potential source of bioactive secreted products, including cytokines and growth factors [62]. Different types of proteases release from cytoplasmic granules after mast cell activation and cleave targets in local tissue environments, but may also play a part in mast-cell-infiltrated lymph nodes or elicit pro-inflammatory reactions in the circulatory system [63]. Chymase is an important component of secretory granules of mast cells. In addition to the synthesis of angiotensin II, chymase is involved in transforming growth-factor-beta activation and cleaves type I procollagen to produce collagen [53]. In addition, mast cells have been recognized as an important component in the development of kidney disease. Mast-cell chymotrypsin can promote the production of angiotensin II, causing increased inflammation in the kidney or other tissues. Inhibition of mast-cell activation and degranulation may have a potential role in reducing inflammation and collagen deposition in AVF failure [63].

This study had several limitations. First, owing to the extensive application of endovascular balloon dilatation surgery, the number of patients undergoing surgical revision AVF has been greatly reduced, and it is extremely difficult to obtain clinical samples of patients. Therefore, we did not obtain sufficient samples for subsequent PCR and WB validation. However, since this dataset only contained first-stage native veins and subsequent AVF failure veins, it was difficult to understand the characteristics of the transcriptomic changes of venous remodeling in a relatively short period after surgery. At present, we attempted to identify the changes in gene expression profiles at different time nodes of venous remodeling by constructing a mouse AVF model in a subsequent study to better reveal the complete process of venous remodeling.

## 4. Materials and Methods

### 4.1. Data Source

The GSE119296 dataset of patients with AVF was extracted from the NCBI Gene Expression Omnibus (GEO) database on 30 June 2022 (https://www.ncbi.nlm.nih.gov/geo/). Raw bioinformatics RNAseq gene counts were obtained from GSE119296 using the GPL18573 platform (Illumina NextSeq 500). A total of 16 native veins and AVFs with failed maturation outcomes were collected from the venous segments of AVFs in hemodialysis patients. Read counts were also converted to FPKMs according to gene length.

### 4.2. Verification for Repeatability of Intra-Group Data

Pearson’s correlation analysis between the two groups was performed to evaluate data repeatability. R was used to draw a heatmap, which shows the correlation between intra-group data. Principal component analysis (PCA) is widely used for gene expression, diversity analysis, resequencing, and other sample clustering based on various variables. Using PCA, we assessed sample relationships and variability in gene expression. For PCA, the analysis was performed using the R package statistics; specifically, the expression profiles were first processed by z-score and further dimensionality reduction analysis was performed using the “prcomp” function to obtain the reduced matrix [64].

### 4.3. Identification of Differentially Expressed Genes

We performed an analysis of the differentially expressed genes (DEGs) using the “DESeq” package in R software (version 4.0.1; https://www.rproject.org/, accessed on 30 June 2022). The significance criteria for identifying DEGs with a low false discovery rate were set as adjusted *p* values < 0.001 and|Log2(fold change)|≥ 1. Three types of DEGs were screened: (1) non-sex-specific DEGs by AVFs vs. native veins (control); (2) male-specific DEGs by AVFs vs. native veins (control); and (3) female-specific DEGs by AVFs vs. native veins (control). The corresponding Venn diagrams, heat maps, and volcano plots were generated using the R package.

### 4.4. Functional and Pathway Enrichment Analysis

Gene Ontology (GO) and Kyoto Encyclopedia of Genes and Genomes (KEGG) enrichment analyses were performed to analyze the functional enrichment of DEGs associated with the biology underlying AVF failure. GO terms with corrected *p* values < 0.05 and KEGG pathways with *p* values < 0.05 were defined as significant enrichments. The R packages ‘ggplot2’, ‘enrichplot’, and ‘GOplot’ were used to create the bubble plot and circle plot of GO and KEGG pathway annotation.

### 4.5. Gene-Set Enrichment Analysis

Gene-set enrichment analysis (GSEA) (http://www.broadinstitute.org/gsea/index.jsp, accessed on 30 June 2022) was used to determine whether the identified sets of genes demonstrated significant differences. The expression levels of all DEGs were analyzed using GSEA_4.0.3. Distinct GO and KEGG gene sets were identified using enrichment maps between the patients in different groups. Normalized *p*-values (*p* < 0.05) and normalized enrichment scores (NESs) were used to determine the functions to be investigated in further analysis.

### 4.6. Analysis of Immune Cells Infiltration Using the CIBERSOFT Algorithm

The CIBERSOFT algorithm determines the relative levels of immune cells within a complex gene expression profile by using the relative levels of different types of immune cells. The ratio of the 22 immune cell subsets was calculated stepwise using CIBERSORT in R according to the mRNA expression of immune signature genes in the AVFs and control native veins, with permutations set to 1000. The Wilcox test was performed to compare the degree of immune cell infiltration between the two groups.

### 4.7. Construction of Protein–Protein Interaction (PPI) Network and Identification of Hub Genes

A protein–protein interaction (PPI) network based on DEGs was constructed using STRING (https://string-db.org/, accessed on 30 June 2022), with the cut-off standard as a combined score >0.8. Subsequently, the PPI networks were exported from STRING and imported into Cytoscape. To identify the hub genes, we ranked all the listed genes according to their degrees calculated in the PPI and co-expression networks. Hub genes in the PPI network were defined as those with the highest degree of connectivity.

### 4.8. Clinical Samples and Immunocytochemistry (IHC)

Native and AVF venous samples were collected from patients at the Wuhan Union Hospital (Wuhan, China). All patients provided informed consent, and the Ethics Committee of Tongji Medical College of Huazhong University of Science and Technology approved this study ([2020]IEC-J(117)). The entire process followed the International Ethical Guidelines for Biomedical Research Involving Human Subjects issued by the Council for International Organization of Medical Sciences (CIOMS) and adhered to the guidelines issued in the Declaration of Helsinki. Paraffin sections of the native vein and AVF venous samples were used for further immunohistochemical validation. Briefly, slices were dewaxed, dehydrated, and repaired using the standard method. The slices were then blocked and treated with the target primary antibody and goat anti-rabbit/mouse IgG. The slices were then dripped with Streptomyces ovalbumin protein labelled with horseradish peroxidase, and the slices were color developed using diaminobenzidine (DAB). Hematoxylin was used as a counterstain. The expression of both COL1A1 and CDK1 were calculated as the integrated optical density (DOI) of the area which was stained yellow-brown by the Image-Pro Plus. The IOD value was divided by the area of the target distribution region to obtain average optical density (AOD). The AOD of both groups were present as the mean ± SEM.

### 4.9. Identification of the Potential Drugs

The Drug Gene Interaction Database (DGIdb) version 3.0.2 (https://www.dgidb.org, accessed on 30 June 2022) is a resource of drug-targeted and sensitive genomes, along with drug–gene interactions. To predict potential drugs or molecular compounds that interact with hub genes, we searched the DGIdb and created a drug–gene interaction list.

### 4.10. Statistical Analysis

Data are presented as mean ± SD. All data were analyzed using GraphPad Prism 8 software (GraphPad Software, La Jolla, CA, USA). Analysis of variance with repeated measures followed by a post hoc Bonferroni’s correction or a Student 2-sample *t* test was used. For all comparisons, a *p* value of <0.05 was considered statistically significant and denoted by * (*p* < 0.05), ** (*p* < 0.01), and NS (not significant).

## 5. Conclusions

Sex differences in AVF failure have been well documented, with females having decreased maturation and patency rates compared to males. However, the underlying mechanism remains obscure to date. Focusing on the bioinformatics analysis, this study shows that coordinated and differential regulation of genes and pathways are associated with sex differences in AVF failure. Immune responses including γδ-T cells and mast cells are activated in female patients while no significant differences were noted in the male group. The present data indicate that several molecular compounds may improve sex-specific AVF remodeling. Taken together, our findings may help guide understanding of sex differences in AVF failure and offer new potential targets for sex-specific treatment strategies.

## Figures and Tables

**Table 1:** Summary of pre-clinical and clinical trials using potential drugs treating AVF failure.

Target	Drug	Sex	Reference
MMP1,MCL1,IL10,PLAU,PIM1	SIROLIMUS	Female	[28]
AURKA,TOP2A,BIRC5,STMN1,CASP3,BRCA2,CDK2,FOS,CDKN2A,PDGFRA,TUBB3	PACLITAXEL	Female	[29]
PTGS2,CASP3,CACNA1A,CACNB4,PLAU,PLAU	CELECOXIB	Female	[30]
TOP2A,NFKB2,BLM,MAPT	QUERCETIN	Female	[31]
JUNB,BIRC5,CDK2,CDK6,CD86,TYMS,SERPINE1,TGFBR3	DEXAMETHASONE	Female	[32]
JUN,IL18,TUBB3,SLC2A4	COLCHICINE	Female	[33]
BIRC5,BCL2L11,MYC,SFN,CDKN2A,CHST1,PDGFRA,SLC2A4,RUNX1	IMATINIB	Female	[34]
MYC,PLAU	CALCITRIOL	Female	[35]
BRCA2	EVEROLIMUS	Female	[36]
CDK2,THBS1,HMGCR,LDLR	LOVASTATIN	Female	[37]
MYC,FCGR3A,CD80	PREDNISOLONE	Female	[38]
PRKAA2	METFORMIN	Female	[36]
SOX10	PACLITAXEL	Male	[29]
MAPT	QUERCETIN	Male	[31]

## Data Availability

All data generated or analyzed during this study are included in this published article.

## Supplementary Materials

The following supporting information can be downloaded at: https://www.mdpi.com/article/10.3390/jcdd10010003/s1, Table S1: Baseline characteristics of the patients; Table S2: Drug–gene interaction network targeting AVF failure

## Abbreviations

AVF: Arteriovenous fistula; ESRD: End-stage renal disease; GEO: Gene Expression Omnibus; DEGs: Differentially expressed genes; GO: Gene ontology; KEGG: Kyoto Encyclopedia of Genes and Genomes; IHC: Immunocytochemistry; RRT: Renal replacement therapies; GSEA: Gene-set enrichment analysis; NESs: Normalized enrichment scores; PCA: Principal component analysis; PPI: protein–protein interaction; CIOMS: Council for International Organization of Medical Sciences; DAB: Diaminobenzidine; DOI: Integrated optical density; AOD: Average optical density; DGIdb: Drug Gene Interaction Database; PCA: Principal component analysis; VSMC: Vascular smooth muscle cell; VDCCs: voltage-dependent calcium channels; ROS: Reactive oxygen species; DCs: Dendritic cells; Treg: T-regulatory.
